# Strain energy-based rubber fatigue life prediction under the influence of temperature

**DOI:** 10.1098/rsos.180951

**Published:** 2018-10-17

**Authors:** Jingnan Zhang, Fengxian Xue, Yue Wang, Xin Zhang, Shanling Han

**Affiliations:** 1 College of Transportation, Shandong University of Science and Technology, Qingdao, Shandong 266590, People's Republic of China; 2 College of Mechanical and Electronic Engineering, Shandong University of Science and Technology, Qingdao, Shandong 266590, People's Republic of China

**Keywords:** temperature, fatigue life, prediction model, strain energy density

## Abstract

Aiming at the problem of the fatigue life prediction of rubber under the influence of temperature, the effects of thermal ageing and fatigue damage on the fatigue life of rubber under the influence of temperature are analysed and a fatigue life prediction model is established by selecting strain energy as a fatigue damage parameter based on the uniaxial tensile data of dumbbell rubber specimens at different temperatures. Firstly, the strain energy of rubber specimens at different temperatures is obtained by the Yeoh model, and the relationship between it and rubber fatigue life at different temperatures is fitted by the least-square method. Secondly, the function formula of temperature and model parameters is obtained by the least-square polynomial fitting. Finally, another group of rubber specimens is tested at different temperatures and the fatigue characteristics are predicted by using the proposed prediction model under the influence of temperature, and the results are compared with the measured results. The results show that the predicted value of the model is consistent with the measured value and the average relative error is less than 22.26%, which indicates that the model can predict the fatigue life of this kind of rubber specimen at different temperatures. What's more, the model proposed in this study has a high practical value in engineering practice of rubber fatigue life prediction at different temperatures.

## 1. Introduction

Owing to their superior ability to good wear resistance and tear resistance, rubber materials are widely used in the automobile industry, for example, the tire, vibration isolator and other parts. For rubber materials, different working temperatures will have different influences on their mechanical properties. The thermal-oxidative ageing reaction of rubber material will accelerate with the increase of temperature, which will lead to the decline of its anti-fatigue property. Under the action of repeated loads, fatigue failure is easily caused, and then potential safety hazards are generated. Therefore, the effective prediction of fatigue life at different temperatures is of great significance for timely replacement and extension of the service life of rubber products. At present, the prediction of rubber fatigue life under the influence of temperature is faced with two major difficulties. On the one hand, it is necessary to carry out the corresponding fatigue test and establish a model to analyse the fatigue characteristics of such parts because of the large variation of ambient temperature and complex loading conditions; on the other hand, there is the difficulty of how to establish the relationship between temperature and fatigue life model to predict the rubber fatigue life at different temperatures.

Many prominent scholars have performed a series of research on the prediction of rubber fatigue life. Fatemi and coworkers [1,2] investigated the prediction method for rubber components, and used an automotive cradle mount as an illustrative component to conduct the analysis including the finite-element analysis, constitutive behaviour representation of the material and fatigue damage parameter. Then, the maximum principal strain was selected as a damage criterion, and Miner's linear cumulative damage rule was used in predicting the rubber fatigue life based on the fatigue crack initiation life and fatigue crack growth life prediction method, respectively. Woo *et al*. [3,4] conducted finite-element analysis and life prediction of the rubber composites and related components by using Green-Lagrange strain as the fatigue damage parameter. Suryatal *et al*. [5] predicted the fatigue life of a railway elastomeric pad by combining the experiment of material properties and using the Mooney–Rivlin model for the finite-element analysis. Then, the maximum first principal elastic strain was selected as the fatigue damage parameter to predict the fatigue life of an elastomeric pad under different compressive loads. Seichter *et al.* [6–8] summarized the advantages of fatigue crack growth theory on the basis of previous research and carried out the uniaxial test to study the fatigue life of rubber. Wang *et al*. [9,10] used the finite-element method to compute three fatigue damage parameters including logarithmic principal strain, Cauchy principal stress and strain energy density for fatigue life. Then, the fatigue life of the rubber vibration isolator in the uniaxial tension state was predicted by using the prediction model based on the least-square method. Shangguan *et al*. [11,12] predicted the fatigue life of the rubber isolator by choosing and analysing different fatigue damage parameters. The fatigue life forecast by the prediction models was compared with the experimental life, which showed that the prediction model which used the effective stress as the damage parameter had the best accuracy. What is more, the model selecting Saintier stress or Luo stress as the damage parameter has a longer fatigue life than the other models.

Fatigue life prediction at different temperatures has a profound significance for rubber components to ensure their reliability and safety. At present, there are a number of studies on the fatigue life of rubber, but there is less research into fatigue life prediction considering temperature factors. Although several scholars have carried out some tests to research the influence of temperature, none of them established a prediction model that chose the temperature as the dependent variable. For example, Mars & Fatemi [13] showed that the fatigue life of rubber material changes with temperature, and besides, the variability of different rubber compounds are different. Neuhaus *et al.* [14] carried out fatigue tests to study the direct influence of chemical and thermal ageing on the fatigue life of natural rubber, and he also studied the various influences of high temperature on fatigue life further. Shangguan *et al.* [15,16] carried out the uniaxial tensile fatigue test on both the specimens with two different hardnesses (45 and 50) and three filled natural rubbers at different temperatures. Then, three kinds of fatigue life prediction models at different temperatures were established using the engineering strain as the damage parameter based on the experimental data. However, the above researchers only studied the fatigue characteristics under the influence of temperature from the perspective of the type of rubber materials and the selection of fatigue damage parameters, and no model was established to demonstrate the relationship between temperature and fatigue life prediction.

Based on the above analysis, the uniaxial tensile fatigue tests of dumbbell rubber specimens are carried out at different temperatures and different loads in this paper. The fatigue life prediction model is established with strain energy as the fatigue damage parameter, and the fatigue characteristics under different temperatures are predicted by the relationship between the model parameters and temperature.

## 2. Experimental set-up

### 2.1. Experiment object selection

**Figure 1.**
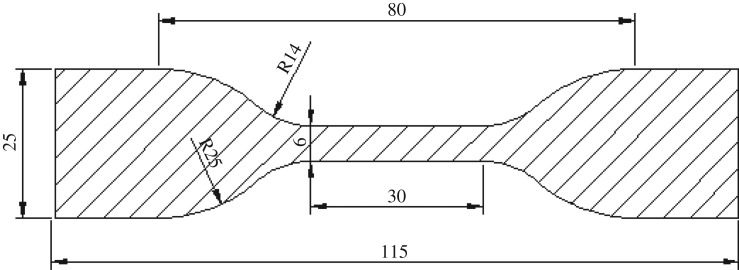
The dimensions of dumbbell rubber test plates (mm).

The uniaxial tensile test is an important means to study the mechanical properties of rubber materials. Based on its simple experimental operation, this study used the uniaxial tensile test to study the mechanical properties of dumbbell rubber specimens. To minimize the influence of rubber specimen thickness on the tensile process, the dumbbell rubber specimen was cut according to the standard in the document GB6672-2001 (plastic film and sheeting—determination of thickness by mechanical scanning) [17]. The distance of the dumbbell rubber test piece is 30 mm and the thickness is 2.5 mm. The width of the middle part of the rubber specimen is 6.0 mm, as shown in figure 1.

### 2.2. Experimental equipment and conditions

The uniaxial tensile experiments at different temperatures were carried out by a 100–100(200) WH oven universal material testing machine whose accuracy of force measurement is ±1%. The dumbbell rubber specimen was placed in the two clamps of the tester to keep the specimen elastic to prevent the specimen from sliding or breaking. The experimental temperature ranges from 20°C to 110°C, and every 15°C was a working condition. Each working condition was tested five times, and the tensile rate was set as 500 mm min^−1^. The data measurement and information acquisition system based on the testing machine could record the tensile data of the specimen in real time, including temperature, initial length *L*_0_, tensile length *L* and stress *σ*.

### 2.3. Test data processing

To minimize the errors in the test process, the measured data were processed in average, namely the average value of the measured uniaxial tensile data was calculated as the initial data. This processing method can reduce the error of test data to a certain extent and improve the accuracy of the recording. The process of strain solution is shown in the following formula:

2.1 $$\varepsilon =\frac{L-L_{0}}{L_{0}},$$

where *L*_0_ is initial length; *L* is tensile length and *ɛ* is the engineering strain.

## 3. Analysis of temperature factors and determination of model parameters

The prediction of fatigue life of rubber materials is divided into two stages: the first stage is the study of crack initiation life based on the continuum mechanics theory, and the second stage is crack propagation life analysis based on fracture mechanics [18,19]. The crack initiation life method using stress or strain to characterize the fatigue life follows the relation between the crack size and the cycle period, which can ignore the influence of a tiny crack on rubber [20]. The crack propagation life method using the stress or strain at the root of the fracture to characterize the fatigue life needs to measure the initial geometry of the crack and the energy release mileage. Because the stress near the crack is concentrated, it is easy to produce a large prediction error [21]. On the basis of observation and analysis of the cracks in the dumbbell rubber specimens, the fatigue crack initiation method was selected as the prediction model [22,23].

### 3.1. Effect of temperature on mechanical behaviour of rubber

**Figure 2.**
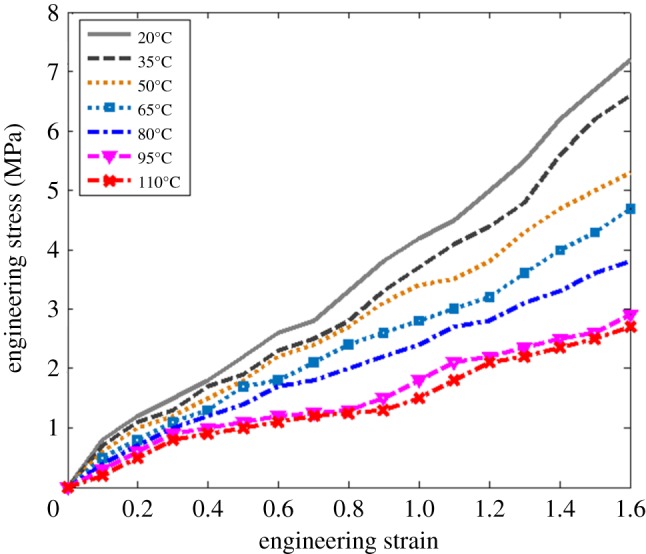
Stress–strain diagram at different temperatures.

The uniaxial tensile test data of dumbbell-shaped rubber specimens at the temperature of 20–110°C were selected, and the stress–strain curves were shown in figure 2 [24]. A phenomenon can be seen from figure 2 that there is an increasing trend of the engineering stress with the increase of the engineering strain at the same temperature, and the trend increases slowly with the increase of temperature. Conversely, there is a decreasing trend of the engineering stress with the increase of temperature at the same strain value, and the trend downward accelerates with the increase of the engineering strain. With the change of temperature, the degradation rate of rubber material is different, and the relaxation degree of rubber material is different, which leads to different stress and strain of gel stretching at different temperatures [25].

There are two main reasons for the above phenomenon: rubber thermal ageing and fatigue damage. The thermal ageing of rubbers mainly refers to the thermal-oxidative ageing reaction, which is the oxidation catalytic reaction initiated by ROOH. The oxidation reaction intensifies with the increase of temperature, which leads to the cracking or structuring of rubber molecules and the destruction of rubber materials [26]. The fatigue failure mechanism of rubber material is that the repeated change of the rubber specimen under load leads to repeated alternating stress below the yield limit and the failure of the rubber specimen [27]. The two kinds of failure interact with each other and cause damage to the fatigue life of rubber at the same time. The reason why the stress and strain of colloid tension are different at different temperatures is that the thermal-oxidative ageing reaction is gentle, the damage degree of molecular chain small, the molecular chain close and the microcosmic gap small when the temperature is low. The molecular movement of rubber materials is accelerated with the increase of temperature, and the thermal oxygen ageing reaction is accelerated. The molecular chains continue to expand and polymerize repeatedly under the load. With the increase of micro-cracks, macroscopic cracks and relaxation degree increase, and irreversible plastic deformation occurs, which leads to the failure of rubber materials.

### 3.2. Selection of fatigue damage parameters

The fatigue damage parameter is a description of the control variable of the load history during the fatigue test. The key to improving the prediction accuracy of fatigue life of rubber materials is to choose the appropriate fatigue damage parameters. What's more, the higher the fitting correlation coefficient of fatigue life and damage parameter, the more accurate the fatigue life prediction will be [12]. Principal strain, strain energy density, Green's strain and octahedral shear strain are usually selected as the fatigue damage parameters to evaluate the fatigue life of rubber materials. Under the action of stress, the corresponding plastic deformation will occur. Plastic deformation is the process of producing residual deformation when the external stress is large and the deformation does not disappear completely with the external stress. Strain energy density is the ratio of energy to size change in the plastic deformation of an object. It not only can describe the distribution of the molecules well but also can analyse the stress and strain well in uniaxial tension tests [28]. Therefore, the strain energy density is selected as the damage parameter to be researched in this study. The formula for calculating the increment of strain energy is:

3.1 dw=σdε,

where *w* is the strain energy density and *σ* is the engineering stress.

The strain energy density under uniaxial tension is calculated by the following formula.

3.2 $$w=\int_{0}^{\varepsilon }\sigma d\varepsilon .$$

The calculation of strain energy density is based on the stress–strain relationship curve under the same conditions, which is fitted by the hyperelastic constitutive model, in order to obtain the material parameters of the hyperelastic constitutive model and establish the stress–strain relationship. The strain energy density can be obtained by substituting the experimental data into the formula (3.2).

### 3.3. Fitting of the experimental data with the hyperelastic constitutive model

Compared to the mechanical properties of metal materials, the stress–strain relationship of rubber materials has stronger nonlinearity. To describe the nonlinear relationship, the hyperelastic constitutive model which can reflect the functional relationship between the deformation of rubber material and the strain energy is introduced. It is generally assumed that the rubber material is isotropic and incompressible when the nonlinear elasticity of rubber is evaluated by the hyperelastic constitutive model, which is to say that the material parameters won't change with the deformation of the colloid [29,30]. The relationship among the strain energy density, the engineering stress and the engineering strain of rubber materials in uniaxial tension can be expressed as follows:

3.3 $$\sigma =2((1+\varepsilon )-{\left(1+\varepsilon \right)}^{-2})\left(\frac{\partial w}{\partial I_{1}}+\lambda^{-1}\frac{\partial w}{\partial I_{2}}\right),$$

where *σ* is the engineering stress; *I*_1_, *I*_2_ are two basic invariant functions of the Cauchy–Green deformation tensor.

Different constitutive models have various performances on the mechanical properties of rubber. At present, there are more than 40 constitutive models including the Mooney–Rivlin model, Yeoh model, Arruda–Boyce model, Ogden model and Neo-Hookean model, which are widely used. According to the existing research, the Yeoh model not only has a relatively simple form but can also predict the material behaviour of complex patterns accurately. In addition, it can be available only through uniaxial tensile experiment data [31,32]. Taking into account the above properties, a Yeoh hyperelastic constitutive model was selected to describe the relationship between strain energy density and deformation tensor in this study. The hyperelastic strain energy density function under the condition of *N* = 3 is defined as follows:

3.4 $$w=\sum_{i=1}^{3}C_{i}\;{\left(I_{1}-3\right)}^{i},$$

where *C_i_* is the material parameter of the mode whose unit is MPa. *C*_1_ is the initial shear modulus under small deformation; *C*_2_ is the softening phenomenon of medium deformation; *C*_3_ is the hardening of material in a state of great deformation.

**Figure 3.**
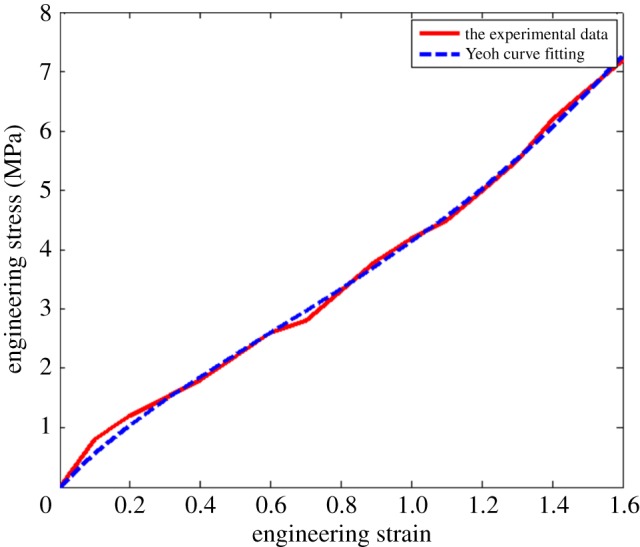
Yeoh curve fitting with different strain ranges at 20°C.

For carbon black-filled rubber materials, ∂*w*/*∂I*_2_ is much less than *∂w*/*∂I*_1_ and approximately zero. The relationship between engineering stress and strain described by the Yeoh hyperelastic constitutive model can be obtained by using formulas (3.3) and (3.4), which is expressed by formula (3.5). On the basis of the above analysis, the material parameters of the Yeoh model are obtained by fitting the stress and strain data and using formulas (3.5) with MATLAB [33,34]. As is shown in figure 3, the Yeoh curve fitting with the strain in the range of 0–1.6 at a temperature of 20°C was illustrated. It can be seen from figure 3 that the Yeoh model curve is similar to the stress–strain curve, and the coincidence degree is relatively high.

3.5 $$\begin{array}{rl} & \sigma =2[(1+\varepsilon )-{\left(1+\varepsilon \right)}^{-2}]\{C_{1}+2C_{2}[{\left(1+\varepsilon \right)}^{2}+2{\left(1+\varepsilon \right)}^{-1}-3]+\\ & 3C_{3}{\left[{\left(1+\varepsilon \right)}^{2}+2{\left(1+\varepsilon \right)}^{-1}-3\right]}^{2}\}. \end{array}$$

The material parameters of the model can be obtained by fitting the curve. The fitting degree of the model can be evaluated by the correlation coefficient *r*^2^ that is defined as the ratio of sum of squares of the regression to total sum of squares. The correlation coefficient *r*^2^ can be expressed by the following formula:

3.6 $$r^{2}=\frac{\mathrm{SSR}}{\mathrm{SST}}=\frac{\sum_{i=1}^{n}w_{i}{\left({\hat{\sigma }}_{i}-{\bar{\sigma }}_{i}\right)}^{2}}{\sum_{i=1}^{n}w_{i}{\left(\sigma_{i}-{\bar{\sigma }}_{i}\right)}^{2}},$$

where ${\hat{\sigma }}_{i}$ is the prediction data; ${\bar{\sigma }}_{i}$ is raw data mean and $\sigma_{i}$ is the raw data.

The correlation coefficient *r*^2^ can represent a fitting accuracy by changing the data. The closer it is to 1, the higher the fitting degree will be, which indicates that the predicted fatigue life of rubber is close to the measured parameters [35]. The model parameters and the correlation coefficient of the fitting are shown in table 1.

**Table 1.** Parameters of Yeoh model at 20°C.

temperature (°C)	*C*_1_ (MPa)	*C*_2_ (MPa)	*C*_3_ (MPa)	*r*^2^
20	1.014	0.03413	0.00266	0.9990

Similarly, the parameters of the model with the strain in the range of 0–1.6 and the fitting correlation coefficient can be obtained at different temperatures, which are shown in table 2. It can be seen from table 2 that the correlation coefficients are close to 1 at each temperature; the change of temperature results in a minor change of correlation coefficient, which indicates that the correlation coefficient is not related to the temperature.

**Table 2.** Parameters of Yeoh model under different temperature conditions.

temperature (°C)	*C*_1_ (MPa)	*C*_2_ (MPa)	*C*_3_ (MPa)	*r*^2^
35	0.9098	0.01724	0.00498	0.9985
50	0.8602	0.01799	0.00112	0.9988
65	0.7825	−0.00960	0.00433	0.9992
80	0.6752	−0.00092	0.00188	0.9994
95	0.5106	−0.00590	0.00223	0.9924
110	0.4576	−0.00599	0.00262	0.9927

According to tables 1 and 2, the overall variation trend of the parameters *C_i_*(*i* = 1,2,3) of the Yeoh model is decreasing with the increase of temperature (ignoring the errors in the fitting process). The decrease of the model parameters *C*_1_ indicates that the shear modulus of the rubber specimen decreases with the increase of temperature. The model parameters *C*_2_ show a negative value in general, and the softening phenomenon occurs in the process of medium deformation of the reaction material. The positive values of the model parameters *C*_2_ at 20, 35 and 50°C during this experiment are due to the fact that under the condition of low temperature, the softening phenomenon of the rubber specimen caused by the small deformation process is smaller, and as the temperature increases, the more obvious the softening phenomenon is. The hardening phenomenon of rubber specimen during the process of extensive deformation is reflected by *C*_3_, the value of which is positive. With the increase of temperature, the hardening phenomenon of rubber specimen caused by large deformation is less obvious [36]. Based on the theory of rubber statistics, the elastic modulus of rubber increases with the increase of temperature and the decrease of average relative molecular weight of the internetworking chain, which accelerates the ageing process of rubber and results in the decline of its mechanical properties. Eventually, the elastic modulus of the material decreases [37].

## 4. Construction of rubber fatigue life model

The fatigue crack initiation is an effective method to predict the fatigue life of rubber materials [38]. It can be solved by the local stress–strain method and the nominal stress method. The nominal stress method is based on the theory of mechanics of materials or elasticity, and the nominal stress of the dangerous point can be calculated by the S–N curve, which is suitable for conditions where the stress level is not high and the load is relatively stable. The local stress–strain method relates the fatigue life of a certain point in the dangerous area. It assumed that the stress–strain history of the dangerous area is the same as that of the smooth specimen, which is suitable for the condition of high stress level and local yield of the material. Based on the fact that the stress range is small and the load is stable, the nominal stress method can be used to solve the fatigue life in this study. The relationship between fatigue damage parameters and fatigue life is established to predict the fatigue life of rubber, which is shown in the following formula. At the same temperature, the relationship between the fatigue life *N*_f_ and fatigue damage parameters *P* is described by the power function, and it can be expressed as follows:

4.1 $$N_{f}=k\cdot P^{b},$$

where *k* and *b* are the model parameters of fatigue crack initiation and *N*_f_ is the fatigue life of rubber, and *P* is the fatigue damage parameter.

### 4.1. Analysis of experimental data

The dumbbell rubber specimens are subjected to uniaxial tensile fatigue tests with the same strain range at 20, 35, 50, 65, 80, 95 and 110°C, respectively. Samples are selected at nine different tensile deformation conditions to establish a prediction model of rubber, and the test data are shown in table 3.

**Table 3.** Uniaxial tensile test data.

process	the engineering strain	process	the engineering strain
1	1.505	6	0.984
2	1.401	7	0.879
3	1.298	8	0.775
4	1.192	9	0.566
5	1.089		

According to the above analysis and the data of tables 1 and 2, the strain energy density of each process at different temperatures can be obtained by using formula (3.2). Strain energy density at different temperatures is shown in table 4. It shows that the strain energy density is positively correlated with the engineering strain at the same temperature. The larger the deformation is, the greater the stored strain energy in the materials will be.

**Table 4.** Strain energy density at different temperatures.

process	temperature						
	20°C	35°C	50°C	65°C	80°C	95°C	110°C
1	4.876	4.329	3.879	3.321	2.862	2.133	1.942
2	4.214	3.728	3.380	2.893	2.505	1.865	1.691
3	3.617	3.194	2.924	2.506	2.177	1.620	1.465
4	3.061	2.699	2.493	2.142	1.866	1.389	1.252
5	2.571	2.267	2.108	1.819	1.586	1.181	1.063
6	2.120	1.871	1.749	1.517	1.323	0.987	0.886
7	1.715	1.515	1.422	1.241	1.082	0.809	0.725
8	1.356	1.201	1.131	0.993	0.865	0.647	0.580
9	0.758	0.675	0.638	0.567	0.493	0.370	0.332

Based on thermodynamic analysis, the elasticity of rubber is caused by entropy change. Under the action of external force, the rubber molecular chain changes from curl state to stretch state, and entropy decreases. When the external force is removed, the molecular chain spontaneously tends to the state of entropy increase due to thermal motion, and the molecular chain restores to the curl state by extension. The strain energy stored in the material decreases with the temperature under the same strain condition [39,40]. This is due to the loss of energy caused by the increasing relaxation of the molecules because of the degradation of the colloid when the temperature rises.

Corresponding to the strain data in table 3, fatigue life was tested at 20–110°C. To eliminate the experimental errors, several tests are carried out under the same conditions. The average of the results was taken as the data of fatigue life, which is shown in table 5. It can be concluded that the rubber fatigue life decreases as the temperature rises under the same strain conditions. The reason why the breakage of the adhesive occurs is owing to the weakening of intermolecular force caused by increased movement of molecules when the temperature rises. In addition, the fatigue life of rubber increases as the strain decreases at the same temperature, the reason being that the intermolecular force weakens with the large strain, leading to the breakage of the molecular chain [41].

**Table 5.** Fatigue life data measured by experiments (unit: number of cycles).

process	temperature						
	20°C	35°C	50°C	65°C	80°C	95°C	110°C
1	36 405	32 551	28 897	22 987	20 062	15 687	10 142
2	45 383	39 324	35 072	29 984	25 548	19 695	14 768
3	61 267	52 876	47 698	42 789	34 330	25 651	18 421
4	83 739	74 026	69 768	55 972	44 938	34 116	24 714
5	112 210	102 193	94 004	75 548	61 148	48 857	34 672
6	156 636	139 555	127 845	102 745	85 299	66 445	45 457
7	205 091	184 213	167 451	157 414	123 623	99 499	67 421
8	316 045	284 756	257 840	212 508	170 297	149 248	104 537
9	1,015,936	942 524	814 675	683 584	584 354	491 642	312 147

### 4.2. Prediction analysis of fatigue life

**Figure 4.**
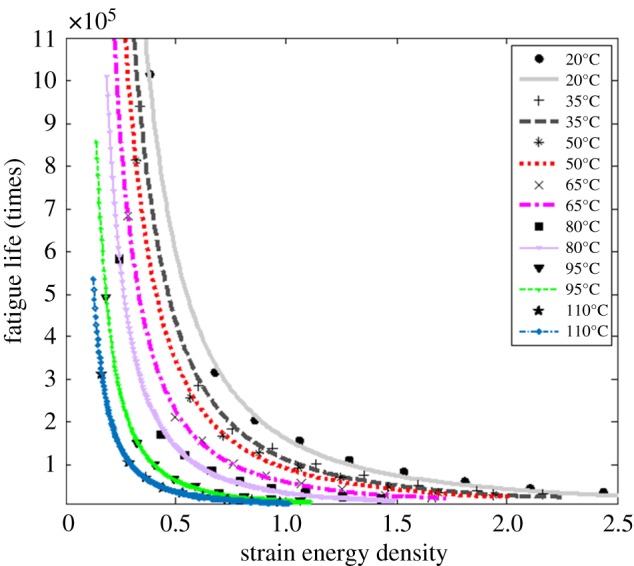
Fitting diagram of strain energy density and fatigue life.

According to the principle of the least-square method, the approximate curve z(x) is obtained from the data points, so that the deviation between the approximate curve and the original curve y=f (x) is minimum, $\min\Sigma_{i=1}^{m}|\phi_{i}|=\Sigma_{i=1}^{m}|z(x_{i})-y_{i}|$. Then, the optimal fitting curve is obtained. Combined with formula (4.1), the model parameters at different temperatures can be obtained by fitting and calculating the strain energy density and the measured fatigue life of the rubber material. The fitting diagram of strain energy density and fatigue life is shown in figure 4 and the model parameters at different temperatures are shown in table 6. It can be seen from figure 4 that the value of fatigue life decreases as the strain energy density increases.

**Table 6.** Model parameters *k*,*b* and the correlation coefficient *r*^2^ at different temperatures.

	20°C	35°C	50°C	65°C	80°C	95°C	110°C
*k*(10^5^)	5.997	4.359	3.441	2.266	1.403	0.6375	0.3677
*b*	−1.894	−1.949	−1.906	−1.942	−2.010	−2.054	−1.937
*r*^2^	0.9985	0.9983	0.9988	0.9987	0.9982	0.9996	0.9998

According to the results obtained in table 6, the experiments at different temperatures correspond to different model parameters *k*, *b*. The model parameters *k* are positive and decrease with the increase of temperature, which means the temperature increases, the coefficient of fatigue life decreases and the fatigue damage degree of rubber specimen increases.

The model parameter *b* is negative. With the increase of temperature, the strain energy density decreases, the cycle number of fatigue life decreases and the model parameter *b* changes accordingly. Its absolute value may indicate the rate of fatigue damage of rubber specimens under different temperature conditions, which increases first and then decreases with the increase of temperature. The reason why the fatigue damage degree of the rubber specimen increases at a faster rate with the increase of temperature, and then tends to slow gradually, is that the strain process leads to the continuous rupture of the rubber molecular chain and the deformation of the rubber with the increase of temperature. And the degree of damage caused by the strain process is more severe. To a certain extent, the rubber is damaged, and the damage rate tends to slow [42].

#### 4.2.1. Fatigue life prediction based on the fatigue crack initiation method

According to the test procedure described above, another group of dumbbell rubber specimens whose compound and material formulations are consistent with those of the dumbbell rubber specimen above was subjected to tensile tests at different temperatures, and the fatigue life data under the condition of engineering strain ε=0.670 were recorded. The predicted fatigue life at different temperatures to the strain value of 0.670 is shown in table 7. Although this method can predict the fatigue life value at different temperatures accurately, it has great defects: different temperatures corresponding to different prediction models, and one prediction model only can predict the fatigue life of rubber material at one temperature. What's more, the experimental data at different temperatures need to be fitted separately to predict the fatigue life of rubber material at corresponding temperatures, and multiple prediction models for rubber can be obtained. The scale of calculation is also too large to be solved as flexibly as the change of temperature.

**Table 7.** Prediction results of the fatigue initiation crack model.

	20°C	35°C	50°C	65°C	80°C	95°C	110°C
prediction value	560 840	513 800	451 470	379 500	316 640	264 620	174 090
measured value	515 746	457 603	414 687	339 767	273 837	209 124	145 873
error	8.74	12.28	8.87	11.69	15.63	26.53	19.34

#### 4.2.2. Prediction model construction with temperature as a variable

It was assumed that the difference of the fatigue life of rubber at different temperatures is related to the model parameters of the fatigue crack initiation method in this study, namely k=k(T), b=b(T). Therefore, the prediction model can be established by combining with formula (4.1).

4.2 $$N_{f}=k(T)\cdot P^{b(T)}.$$

**Figure 5.**
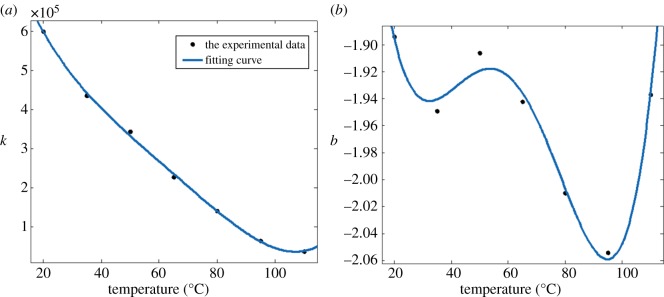
(*a,b*) Fitting of model parameters and temperature.

The relationship between model parameters *k*, *b* and the temperature was fitted by the least-square polynomial method with temperature as a variable, as shown in figure 5*a*,*b*. Through the analysis of scattered plot, the nonlinear relationship between the model parameters *k*, *b* and temperature was fitted by a polynomial curve. Meanwhile, the magnitude of error was judged by fitting correlation coefficient. The relationship between the model parameters *k*, *b* and temperature was determined to be the fourth-degree polynomial through multiple analysis and repetitive fitting. The fitting correlation coefficient is 0.990, 0.983, respectively, which can indicate that the fitting is great. Then, the corresponding polynomial coefficients are obtained, respectively, and the fitting equations of the model parameters and the temperature are shown in figure 5*a*,*b*. The nonlinear equations of the model parameters *k*, *b* and temperature are as follows:

$$k=0.01914x^{4}-4.87x^{3}+466.7x^{2}-2.642\times 10^{4}x+9.758\times 10^{5}$$

$$b=7.183\times 10^{-8}x^{4}-1.731\times 10^{-5}x^{3}+0.001419x^{2}-0.04717x-1.393$$

## 5. Example verification

The fatigue life data of another group of rubber specimens at different temperatures under the engineering strain ε=0.670 are selected to verify the fatigue life prediction model of temperature influence constructed in this paper. Aiming to measure the accuracy of the prediction model, the average relative error is calculated, which is shown in table 8. The error of fatigue life between the experimental and predicted values is small. Among them, the maximum error is 22.26%, which is within the acceptable range. For rubber material, the acceptable error range of the fatigue life prediction is within 100% [9,43]. Therefore, the fatigue life predicted by the prediction model is close to the experimental one.

**Table 8.** Prediction results of a prediction model with temperature as a variable.

	20°C	35°C	50°C	65°C	80°C	95°C	110°C
prediction value	559 390	521 450	436 950	392 110	314 530	255 680	174 810
measured value	515 746	457 603	414 687	339 767	273 837	209 124	145 873
error	8.46	13.95	5.36	15.40	14.86	22.26	19.83

From the comparison of the results of fatigue life prediction shown in tables 7 and 8, it can be seen that the predicted values of the two prediction models are close, and both of the average relative errors are within the acceptable range. There is a decreasing trend of the prediction results of two prediction models as the temperature increases. However, it is more complicated to predict fatigue life at different temperatures by the fatigue crack initiation method, and different temperature conditions need to be solved by experiments with temperature as a variable. Compared with this, the prediction model based on temperature can predict fatigue life at different temperatures and strain energy density, and the prediction method is more flexible and concise. Therefore, the prediction model based on temperature is accurate and effective because of its small error between the predicted fatigue life value and the fatigue crack initiation method.

## 6. Conclusion

In this paper, the prediction model proposed with temperature as the dependent variable provides a means of application for prediction of rubber fatigue life under the influence of temperature. It can predict the fatigue life of rubber materials at different temperatures effectively. The error between the predicted fatigue life and the measured fatigue life is within the acceptable range. Based on the experimental data and model validation, the following conclusions can be drawn.

1. The fatigue life prediction model of uniaxial tension can be well established by a power law, which selects the strain energy density as the fatigue damage parameter. The fourth-degree polynomial relationship between the parameters of the fatigue crack initiation model and the temperature is established to predict the fatigue life at different temperatures flexibly. The correlation coefficient of the fitting model can reach about 0.99.
2. The fatigue life prediction model based on the tensile fatigue test data of dumbbell specimen at different temperatures can be used to predict the tensile fatigue life of this kind of rubber specimen. The maximum average relative error between predicted and measured fatigue life is only 22.26%. According to the method described in this paper, the fatigue life prediction model of rubber material at different temperatures can be obtained by testing dumbbell-shaped test pieces to predict the fatigue life of rubber products in engineering practice. It can shorten the experimental time and reduce the development cost; what's more, it also has high use value.

## Data accessibility

The datasets supporting this article are included as part of the published manuscript as tables 1–8.
